# Considerations and guidelines for photic measurement in field studies of the physiological effects of light

**DOI:** 10.1038/s44323-026-00110-3

**Published:** 2026-10-07

**Authors:** Sara C. Bessman, Elizabeth M. Harrison, Andrea Wilkerson, Michael Herf, Morgan Pattison, Celine Vetter, Gena L. Glickman

**Affiliations:** 1 Uniformed Services University of the Health Sciences, Bethesda, MD USA; 2 Henry Jackson Foundation for the Advancement of Military Medicine, Inc., Bethesda, MD USA; 3 Pacific Northwest National Laboratories, Portland, OR USA; 4 f.lux Software LLC, Los Angeles, CA USA; 5 SSLS, Inc., Johnson City, TN USA; 6 University of Colorado Boulder, Boulder, CO USA

**Keywords:** Neuroscience, Optics and photonics, Physiology

## Abstract

Guidelines for light measurement in laboratory studies of the non-visual effects of light have recently been put forward. Yet, outside of controlled environments, unique challenges necessitate additional consideration and modifications to those recommendations. The purpose of this paper is to build on prior work by outlining pre-ocular, ocular, and post-ocular factors and associated considerations that influence photic exposure and the physiological effects of light in the field. Practical constraints of applying laboratory measurement guidelines and tools and strategies for light measurement in field-based work are discussed in detail, including the trade-offs of different approaches. A standard operating procedure for measuring light in field studies is provided, with particular attention to managing sources of variability, along with an example that applies the procedure to a recent study. Finally, future directions and remaining challenges of photic measurement during field studies of sleep, circadian rhythms, and other non-visual effects of light are identified.

## Introduction

Historically, lighting has been designed primarily to support the human visual system. Yet, there is a growing appreciation for how the effects of light extend well beyond illumination for visual perception. In addition to vision, light entering the eye significantly influences a range of physiological and behavioral processes, including the circadian timing system, neuroendocrine regulation, acute alertness, and mood^1^. An increased understanding of these additional effects of light has driven the development of novel lighting strategies aimed at supporting both visual and non-visual photic responses in domestic and workplace settings, with the goal of improving health and safety outcomes^2^. Parallel advancements in lighting technologies and an increase in non-standard schedules have introduced greater variability in human photic exposure patterns. For example, light-emitting diodes (LEDs) can differ considerably in their physical properties, and exposure to light can vary considerably both within and between individuals.

Studying natural photic exposure patterns and evaluating the efficacy of lighting interventions requires precise and consistent description and quantification of light input, an undertaking that poses unique challenges in field settings. Inadequate measurement and reporting of light stimuli can result in misleading findings, hinder replication, limit comparability across studies, and ultimately slow the development of effective, integrated lighting solutions. While lighting standards and guidelines around vision have existed for decades, consensus-based recommendations have now introduced novel lighting metrics and standards that are specific to non-visual physiological effects of light^3–6^; however, implementing and validating these recommendations in real-world environments requires obtaining light measurements that reflect actual human photic exposure.

In 2019, a set of guidelines was proposed for measuring and reporting light in laboratory studies examining the physiological effects of photic exposure in humans^7^. A subsequent consensus-based framework built upon these recommendations, establishing a standardized process for obtaining light measurements in controlled laboratory settings^8^. These publications addressed a critical need for rigorous characterization of light in laboratory research. While many of the underlying principles can be extended to field studies, light measurement in applied settings poses additional complexity that warrants further consideration.

The purpose of this paper is to build on prior work by outlining a myriad of environmental factors that impact light responses in the real world and proposing strategies for measuring and reporting photic exposure in field-based studies. We begin by providing background on how light information travels through the environment and neural systems, from its source to the physiological responses it elicits, and describe key influences (both direct and indirect) on photic exposure and the physiological effects of light. We then summarize laboratory methods used to manage these variables, highlighting those that may be adapted for field research and discussing the practical constraints of applying them outside controlled environments. Building on this foundation, we propose tools and strategies for field-based light measurement, including standard operating procedures and associated examples, with particular attention to managing additional sources of variability. Finally, we identify key challenges that remain to be addressed.

## Factors that influence photic exposure and the physiological effects of light

**Fig. 1:**
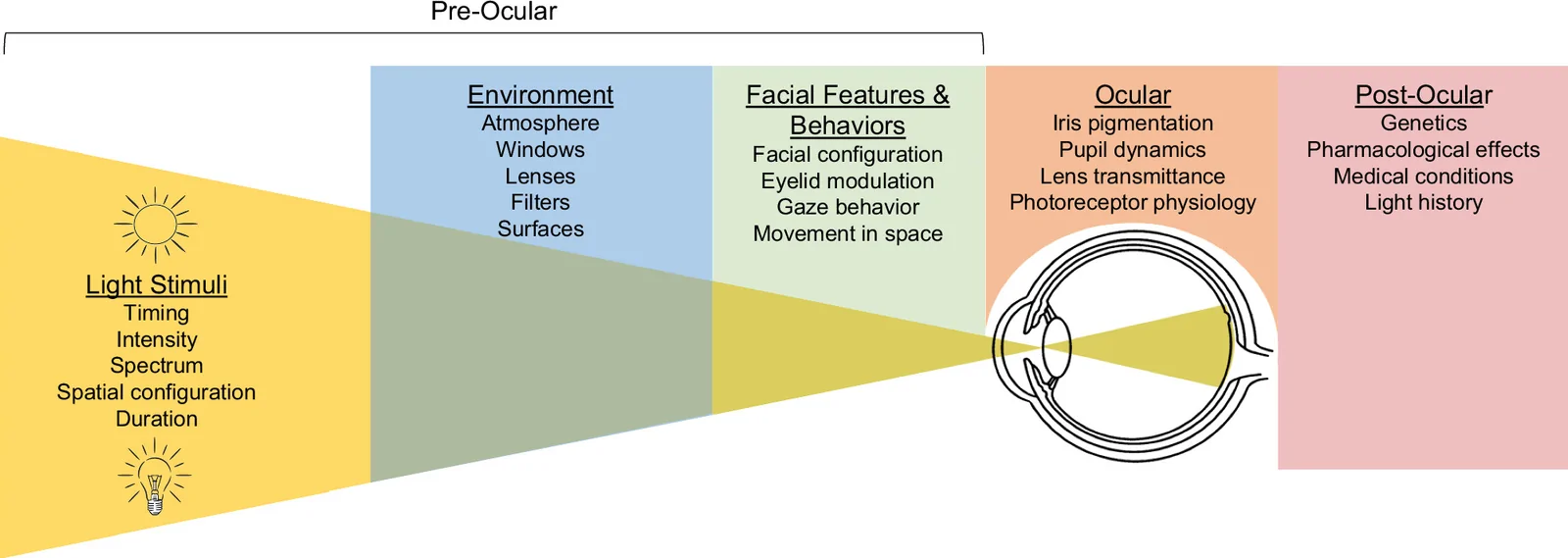
Characteristics that influence the physiological effects of light in humans. In this schematic, the route by which light information travels from its source, through the environment, and within human systems is depicted, including the pre-ocular (yellow, blue, and green), ocular (orange), and post-ocular (red) factors that alter it along the way.

The eye serves as the gateway through which light influences a host of physiological processes, and a wide range of factors can modulate those effects. The neural pathways associated with non-visual functions have been well-described, partly via rigorous studies of how the physical properties of a light source (e.g., intensity, wavelength) affect responses. In practice, however, those physical properties may be altered even before light reaches the eye due to pre-ocular elements (e.g., environmental context, individual behaviors, facial anatomy), along with additional modulation at ocular and post-ocular levels (see Fig. 1).

### Pre-ocular

#### Light stimuli

The rationale and standardized methods for measuring and reporting the physical characteristics of sources of light in the lab have been detailed elsewhere^7,8^. Yet, ambient lighting in the field is typically much more complex and variable, often including multiple different sources of light that are dynamic in nature. Non-visual physiological responses to even just a single light source have been shown to depend on timing, intensity, spectrum, spatial configuration, and duration^1^.

Where quantitative estimates are available, they derive predominantly from controlled laboratory studies under specific exposure conditions. We include a small number of examples solely to illustrate the range of reported physiological effects under those conditions. These values should not be interpreted as representative of real-world exposures or as supporting a universal ranking of the relative importance of different light characteristics. Although some characteristics of light generally exert larger physiological effects than others, the magnitude of the effect associated with any one characteristic depends on the combination of other light characteristics and the exposure context, which makes it difficult to definitively quantify the relative importance of each in practice.

#### *Timing*

Among the factors that influence circadian regulation, the timing of photic exposure is unique in that it affects both the magnitude and direction of phase shifts. Light exposure early in the biological night produces phase delays, whereas the same stimulus late in the biological night or early in the morning produces phase advances. Phase response curves (PRCs) describe this relationship between circadian timing and phase shifting. In a highly controlled laboratory study, a PRC was generated using a 6.5 h pulse of bright white light (10,000 lx), demonstrating maximal phase delays and advances approximately 3–4 h before and after the core body temperature minimum (CBTmin), respectively, with little or no phase shifting near the CBTmin or during the biological day^9^. Although the overall shape of the PRC is highly reproducible, the magnitude and exact timing of phase shifts vary with stimulus intensity, spectrum, duration, prior light history, and individual circadian physiology. PRCs from studies using lower-intensity or intermittent light exposures exhibit a similar Type 1 waveform but with smaller maximum phase shifts, illustrating that although the magnitude of the response depends on the characteristics of the light stimulus, the temporal dependence remains remarkably consistent^10–12^. In addition to phase shifting, other physiological effects, such as acute alerting properties, appear to be more sensitive to light at night versus the day, though this may be due to greater capacity for change from baseline during those hours (i.e., people are sleepier at night)^13^.

#### *Intensity*

At the same circadian phase, the physiological effects of light exhibit an intensity-dependent response, with increasing photic intensity producing progressively larger effects until saturation is reached. This relationship is typically described by a sigmoidal dose-response curve, characterized by a threshold, a half-saturation constant (ED50), and a maximum response. In a landmark laboratory study using a 6.5 h pulse of polychromatic white light, ED50 values were approximately 100 lx for circadian phase shifting and 165 lx for melatonin suppression, with responses saturating at approximately 500 and 1000 lx, respectively^14^. A subsequent within-subject study reported a lower average ED50 for melatonin suppression (~25 lx) but also demonstrated striking interindividual variability within a reasonably homogenous population of young adults, with 50% suppression occurring at ~6 lx in the most sensitive participant and ~350 lx in the least sensitive^15^. Together, these studies illustrate that both the range of responsiveness and the interindividual sensitivity can vary considerably. In addition, many of the factors described below will further influence the absolute values of key dose response parameters.

#### *Spectrum*

Comparing dose-response curves across wavelengths yields action spectra, which describe the spectral sensitivity of non-visual responses to light. Laboratory studies of non-visual physiological effects of light have consistently identified a peak sensitivity in the short wavelength region of the spectrum (470–500 nm)^16–20^, corresponding closely to the peak absorbance of melanopsin^21^. In one human action spectrum study using monochromatic light in participants with pharmacologically dilated pupils, ED50 values for melatonin suppression ranged from ~2 lx at 464 nm to ~400 lx at 620 nm, representing a ~200-fold difference in sensitivity. However, these experimental conditions likely exaggerate spectral differences relative to typical real-world exposures, where light sources are predominantly polychromatic and pupils are freely responsive. For example, under polychromatic white light of similar photopic illuminance (~130 lx), blue-enriched lighting (~17,000 K) produced only approximately twice the predicted biological potency relative to the standard white light condition (4000 K), resulting in increased melatonin suppression and subjective alertness but no difference in circadian phase delays^22^. Spectral measurements can be used to derive α-opic quantities that estimate stimulation of each cone class, rods (rhodopic), and melanopsin-containing ipRGCs (melanopic), providing biologically relevant measures of light exposure^3,4^.

#### *Spatial configuration*

While the spatial arrangement of photic stimuli can impact the intensity and spectral quality of light entering the eye (and thus biological potency), the regions of the visual field that ultimately receive light input can also alter physiological responses directly^23^. This is especially relevant to field studies, where illumination across the visual field is often non-uniform and relative position varies over time. In laboratory studies using polychromatic white light and pharmacologically dilated pupils, illumination of the inferior retina produced approximately 2–3-fold greater melatonin suppression than illumination of the superior retina, with similar differences observed between nasal and temporal retinal stimulation^24–27^. Likewise, binocular exposure generally produces greater melatonin suppression than monocular exposure for the same photon dose^28,29^. Although these findings suggest that the region of retinal exposure influences the biological effectiveness of light, they have been demonstrated under relatively limited laboratory conditions and may interact with other characteristics of the light stimulus. It has been proposed that a spatial weighting function may ultimately be a useful addition to existing spectral weighting approaches^30^.

#### *Duration*

The duration of photic exposure also influences physiological responses. Even brief exposures, including single 15 s or 2 min pulses of bright polychromatic light (~9500 lx), can elicit significant melatonin suppression and phase shifts when administered at appropriate circadian times^31,32^. However, increasing exposure duration yields diminishing returns. In one study, a 12 min exposure was more than five times as efficient per minute at producing circadian phase delays as a 4 h exposure, with half-maximal phase shifting occurring after approximately 1.8 h^33^. Although this non-linear relationship appears robust, the magnitude of the effect of exposure duration may depend on wavelength, circadian timing, and other characteristics of the light stimulus^34,35^.

#### Environment, physical features, and individual behaviors

Beyond the light source itself, additional factors can alter the physical properties of light entering the eye, and these are not always easily controlled in field studies. They include environmental characteristics, facial features, and behavioral patterns. Environmental characteristics such as geographic location, azimuthal orientation, atmospheric conditions, windows and skylights, and surface finishes within a space may all influence photic intensity, absorption, scattering, transmittance, and reflectance^1,30^. For example, greater intensities of sunlight might be experienced with higher elevations, lower latitudes, and clearer days. Atmospheric pollutants, smoke, and humidity levels also affect the scattering and absorption of light particles; however, when outdoors, time of day will typically have a greater impact. When bringing outdoor light indoors, windows can vary in their transmittance properties, based on their size, location, transparency, tint, and coverings, all of which can influence photic intensity and spectrum. Surface colors and finishes (e.g., white or glossy surfaces) can further influence light reflectance and diffusion, ultimately shaping how much light reaches the eye.

**Fig. 2:**
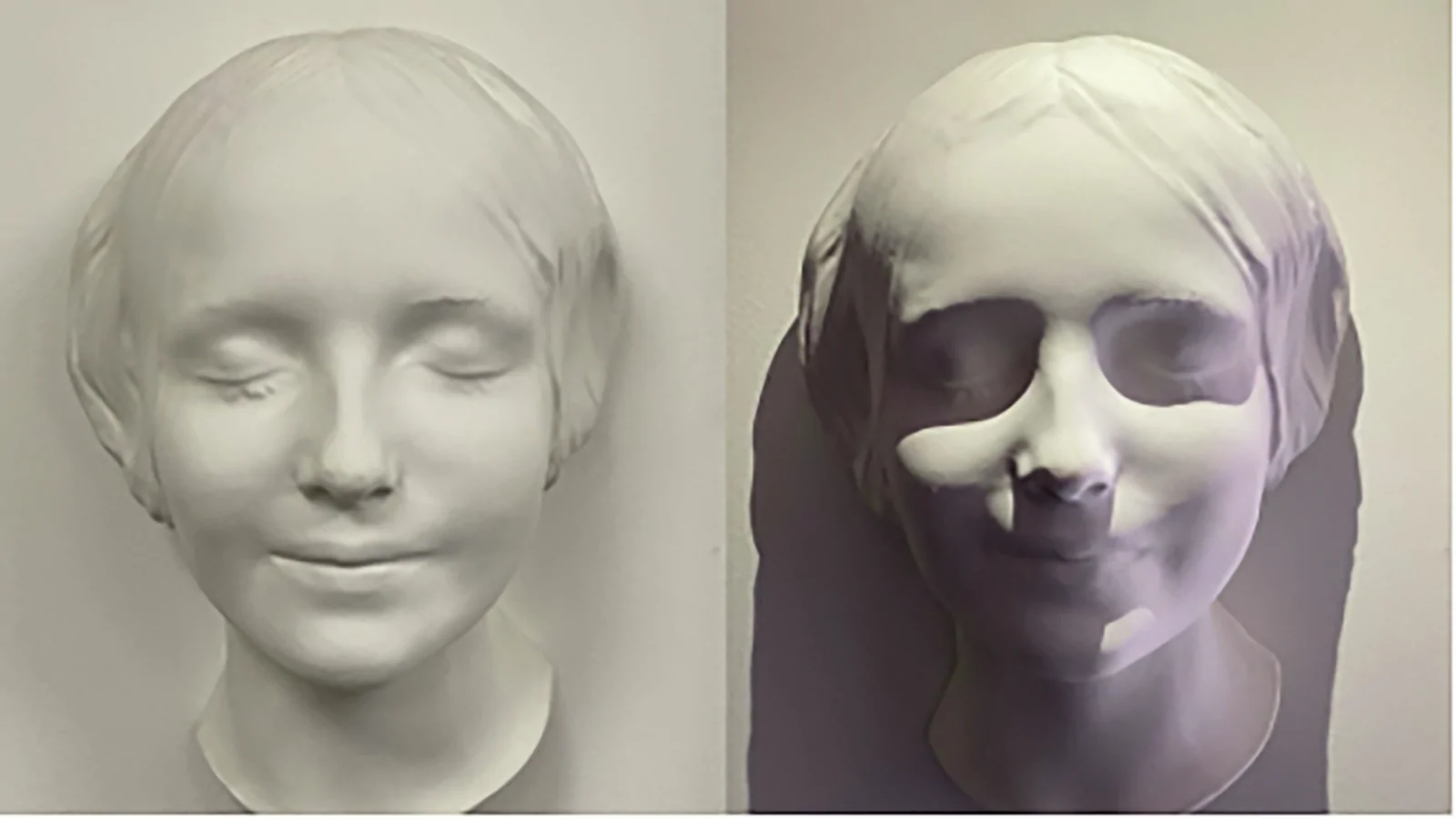
Effect of spatial arrangement of photic stimuli relative to an observer. This model demonstrates how the spatial arrangement of a light stimulus can make a significant difference in the photic dose at the level of the eye or meter sensor. Photographs were taken by Michael Herf and first presented at the Department of Energy Solid-State Lighting Program R&D Workshop in January 2020.

Beyond these environmental factors, individual differences in physical height and facial configuration and morphology, as well as certain behaviors, will further affect the amount of light reaching the eye^36^. These physical features alter occlusion, shading, and the geometry of incident light at the corneal surface^37^. Additional morphological differences may influence the intensity of light entering the eye, including characteristics such as brow ridge size and length; density of eyelashes (natural or prosthetic); and depth of eye sockets^37^ (Fig. 2). Individual photic exposure patterns may also be affected by behaviors such as squinting; direction of gaze; tilt of head; blinking; use of accessories (e.g., hats, helmets, sunglasses, or progressive lenses); deliberate filtering devices and software applications (e.g., spectral-filtering glasses, smartphone light filtering apps); and movement within and between spaces. For example, behavioral responses to glare, such as changes in head position, gaze direction, or partial eyelid closure, may reduce retinal light exposure under very bright conditions, in some cases resulting in lower effective photic input than under dimmer but less aversive lighting environments. Even small postural or positional changes can significantly shift the intensity and spatial distribution of retinal light input, while changes in viewing angle can alter the relative proportion of the retinal regions that are stimulated.

### Ocular

Once the photic signal has entered the eye, factors such as iris color, pupil dynamics, and lens health can further influence physiological responses to light. Individuals with a lighter-colored iris tend to be relatively more sensitive to light as compared to those with darker-colored eyes^38^. The muscles controlling the iris are also important for dilating or constricting the pupil, which determines how much light will reach the retina. Mydriatic agents and other medications (such as the use of pilocarpine drops for presbyopia) can alter pupil dynamics, which can in turn influence physiological effects of light^39^. Dose response curves have demonstrated a ~38% reduction in sensitivity to short wavelength light for melatonin suppression when the pupil is freely constricting versus a pharmacologically dilated condition within the same participants (Glickman, unpublished data).

Independent of pupillary dynamics, there are developmental changes in ocular physiology that can also affect individual responses to light. For example, in young children (e.g., preschool age), there appears to be significantly increased sensitivity to light for both phase shifting and melatonin suppression, with close to maximal responses at as low as 5 lux^40,41^, though this begins to attenuate through puberty^42^. By older adulthood, physiological responses to light are typically reduced^43–45^, likely due in part to a natural yellowing of the lens^45–47^ and reductions in pupil size^48^. For example, one study found that lens transmittance for short-wavelength light decreased from approximately 70% at age 20to 25% at age 70, reducing the amount of biologically effective light reaching the retina^45^. As a result, higher environmental light levels may be required to elicit comparable non-visual responses in older adults.

As light reaches the retina, the classical photoreceptors (rods and cones), along with melanopisn-containing ipRGCs, transduce light for visual and non-visual physiological effects, though the latter responses are primarily mediated by ipRGCs^49^. Each photoreceptor type contains unique opsins that absorb and transduce light to varying degrees within the visible spectrum (~360–770 nm), and human melanopsin has a peak spectral sensitivity of approximately 480 nm^21^. Melanopsin-containing ipRGCs integrate light information more slowly, and the response is more sustained as compared to the classical photoreceptors^50,51^. Importantly, ipRGCs interact with the rod and cone photoreceptor pathways^19,52,53^; however, the way in which these pathways function, both separately and interdependently, to influence biological processes in mammals remains an active area of study^54,55^. When any component of this system is compromised (e.g., ocular health conditions such as certain forms of blindness, photoreceptor deficits, photosensitivity disorders), physiological responses to light may be impaired or lost completely^19,56–58^.

### Post-ocular

Whereas pre-ocular and ocular factors primarily determine the quantity and distribution of photic input reaching the retina, post-ocular pathways determine how that signal is transmitted, integrated, and transformed into downstream physiological and behavioral responses.

Retinal photic signals are transmitted to various brain regions through both visual and non-visual pathways. Light information follows the visual neural pathway via the optic nerve, chiasm and tract to the lateral geniculate nucleus (LGN), intergeniculate leaflet (IGL), and ultimately, visual cortical regions of the brain. Light input driving non-visual physiological responses, such as circadian and neuroendocrine regulation, travels a separate route to the suprachiasmatic nuclei (SCN) via the retinohypothalamic tract (RHT). The SCN, which is the central circadian pacemaker in mammals, projects to numerous other brain regions, including the septum, thalamus, midbrain, and spinal cord. Certain neurological conditions (e.g., Alzheimer’s disease) can result in circadian disturbances that are thought to be due to changes in the integrity of the SCN^59,60^. Both non-visual and visual effects of light are also influenced by indirect pathways between the LGN/IGL and SCN^1^. Additionally, ipRGCs have direct projections to other brain regions outside of the SCN that contribute to pupil constriction, sleep, and emotional regulation^61–64^. Thus, if any of those neural substrates are compromised or altered, it may influence the physiological effects of light.

A variety of additional contextual factors can further modulate non-visual responses to light. Biological characteristics, such as genetics, can influence circadian period as well as chronotype (a phenotype used to describe diurnal preferences), and therefore, phase^65^. There are also significant individual differences in terms of sensitivity to light, with reports of a greater than 50-fold difference in melatonin suppression across a relatively homogenous population of healthy young adults^15,66^. Furthermore, pharmacological agents and health can amplify those differences. For example, certain SSRIs (e.g., citalopram) have been shown to increase melatonin suppression and may affect other physiological responses to light as well^67^. In addition, there are medical conditions (e.g., affective disorders, sleep disorders) commonly associated with circadian disturbances that can impact photic exposure patterns via alterations in sleep and wake times^68^.

Even prior photic exposure (“light history”) can significantly modulate subsequent physiological responses to light. Studies have shown that the relative photic intensity in the hours and days leading up to a test pulse of light can modulate the magnitude of the response observed, for both melatonin suppression and phase shifting in humans^69,70^. There is also evidence that prior day length (i.e., photoperiod) may influence human circadian physiology, such as patterns of melatonin secretion in some individuals^71–76^. Research in model systems has found a relatively greater sensitivity for phase resetting by light under winter photoperiods (short days) as compared to summer (long day) conditions^77,78^. Consistent with that, humans demonstrate increased light-induced phase shifts when they are made to sleep a relatively longer duration, with a resultant shorter photoperiod^79,80^. Although the influence of light history is well-supported, the specific duration, timing, and physiological responses most affected remain areas of active investigation.

## Unique considerations for field studies

The preceding sections described factors that influence the physiological effects of light, most of which have been characterized under controlled laboratory conditions. Although isolating individual variables in the lab is essential for understanding underlying mechanistic pathways, field studies must instead contend with the simultaneous interaction of these factors under ecologically variable conditions. Consequently, the central challenge in applied lighting research is often not identifying the relevant determinants of photic responses, but accounting for the ways in which environmental variability, individual behaviors, biological heterogeneity, and practical implementation constraints collectively shape effective light exposure in real-world settings. In addition, laboratory protocols are typically optimized to maximize experimental control and isolate specific physiological responses, whereas applied lighting interventions must balance non-visual effects with visual performance, comfort, usability, compliance, and feasibility. These translational considerations introduce methodological complexities that are difficult to standardize across participants and settings, but which are critical for interpreting and applying findings outside of the laboratory.

### Light stimuli

In controlled experiments, photic stimuli can be precisely specified in terms of spectral power distribution (SPD), intensity, spatial distribution, and temporal characteristics, enabling tight control over light exposure^7,8,81^. In contrast, real-world lighting environments are typically composed of multiple dynamic light sources whose properties vary across space and time.

Laboratory studies also frequently employ monochromatic or narrow-band stimuli to isolate wavelength-dependent physiological responses or maximize photic efficiency within targeted spectral regions^16–18,82–85^. While these approaches are sometimes essential for mechanistic inference, they do not fully capture the spectral and spatial complexity of real-world lighting environments. Because applied settings must also support visual performance, comfort, and usability, lighting interventions in the field typically rely on polychromatic light sources, including daylight, LEDs, fluorescent, and incandescent lighting, sometimes with targeted short-wavelength enrichment^86–92^.

Current “white light” products often exhibit relatively similar spectral characteristics, despite differences in source technology. As a consequence, variation in photic intensity across everyday environments typically exerts a greater influence on cumulative biological impact than spectral quality. Differences in illuminance between indoor, outdoor daytime, and nighttime settings can span orders of magnitude, frequently outweighing the comparatively modest spectral differences among most common electrical light sources in use today. Therefore, outside the lab, photic dose is often shaped more strongly by exposure context and duration than by fine-scale spectral variation alone.

Lighting technologies are evolving rapidly, however, with increasing emphasis on the design of systems that optimize both visual and non-visual physiological effects of light. Modern lighting technologies have substantially expanded the range of controllable lighting characteristics available for both research and application. LEDs, in particular, provide improved energy efficiency while allowing flexible tuning of spectral composition, intensity, temporal modulation, and spatial distribution, including narrow-band or multi-peak SPDs spanning much of the visible spectrum. Most white LEDs achieve broad spectrum emission through a short-wavelength “blue pump” (~440–460 nm) combined with broader phosphor-based emissions in the yellow and red portions of the visible spectrum, resulting in a similar spectral profile. However, as these technologies advance and become more widely implemented in applied environments for diverse applications, spectral differences between lighting systems will likely become increasingly varied and complex.

Accurate characterization of light exposure in applied settings is therefore becoming considerably more challenging. In the field, multiple co-occurring light sources may differ in intensity, spectrum, spatial distribution, and temporal stability, with additional variability introduced by participant movement and changing environmental conditions^30,93^. Consequently, a single measurement of a space may not adequately represent individual photic exposure over time, and repeated environmental assessments or continuous monitoring may be necessary to estimate effective retinal dose.

### Environment

Environmental characteristics introduce significant heterogeneity in real-world photic exposure that is typically minimized in laboratory settings. Built spaces vary widely in their capacity to modify the distribution, intensity, and spectral composition of light reaching the eye due to differences in access to daylight, architectural design, surface properties, and material composition^1,30^. These factors collectively shape both the spatial and temporal structure of ambient light exposure.

Geometric features, such as window size, orientation, placement, and the presence of skylights and/or coverings, strongly influence the intensity and directionality of daylight. Material and surface properties, including color and gloss affect reflectance and further modulate how light is absorbed, scattered, and redistributed within a space. In addition, operational and contextual factors such as occupancy patterns, furniture layout, and intended use within a space can alter exposure conditions over time in ways that are not typically standardized in applied settings, though they are often controlled for in the lab.

Importantly, these environmental determinants are also not static in the real world. Rather, they fluctuate across time of day, season, weather conditions, and patterns of human activity, making it difficult to maintain consistency or fully characterize lighting conditions across participants or study periods. As a result, a single description of an environment may be insufficient to capture the effective photic exposure experienced within it.

### Human attributes, behaviors, and individual characteristics

Human attributes and behaviors introduce inter- and intra-individual variability in both photic exposure and physiological responses to light. Laboratory studies typically reduce this variability through extensive standardization procedures, whereas field settings must accommodate a broader range of uncontrolled anatomical, behavioral, and physiological differences.

As discussed above, anatomical characteristics influence the amount of light entering the eye^37^. To minimize these effects in the laboratory, light sources are typically fixed in position and distance, and head and body posture may be standardized as well. Some protocols further monitor or constrain gaze direction and eye state during exposure (though squinting remains hard to control), and specialized systems such as ganzfeld domes with matte reflective interiors can be used to produce more spatially uniform retinal illumination. In applied environments, however, photic exposure is continuously modulated by natural behavior. Movement within the environment, in particular, significantly alters photic input in spaces with directional or proximal light sources.

Beyond geometry and behavior, ocular and physiological factors further shape effective photic dose. Under laboratory conditions, pupil size may be pharmacologically controlled or explicitly measured to estimate retinal illuminance (e.g., via trolands). In real-world settings, pupil dynamics instead vary continuously in response to ambient light, influencing retinal light flux and downstream physiological responses. Pharmacological agents and health conditions can further modify this response; for example, some medications induce mydriasis, increasing retinal irradiance, whereas others promote miosis, reducing it^94,95^. While such factors may be controlled through pharmacological dilation of participant eyes and/or via study exclusion criteria in laboratory research, doing so in field studies reduces feasibility and generalizability.

Individual differences in light exposure history further contribute to variability in physiological responses. Prior light exposure can modulate both acute and circadian responses to subsequent light stimuli^69,70^, although the relevant temporal window and most sensitive response domains remain incompletely defined. Laboratory protocols often attempt to standardize this by enforcing predefined sleep-wake schedules and/or periods of dark adaptation; however, such controls are usually impractical in applied work.

Finally, stable biological and demographic characteristics, such as age, ocular health, and genetics contribute additional variability. Although laboratory studies may use screening criteria to reduce heterogeneity, these restrictions can limit generalizability to real-world populations. In addition, subjective differences in discomfort sensitivity (e.g., glare and flicker) and illuminance preferences contribute to variation in both exposure behavior and tolerance^96^. More recently, there has been increased emphasis on incorporating such implementation-relevant factors into field-based lighting research to better capture how individuals experience and engage with light^97^.

Taken together, these considerations highlight the difficulty of fully standardizing photic exposure in applied settings. Rather than attempting to control all sources of variability within a single study, applied research typically requires prioritization based on research questions, available measurement tools, feasibility constraints, and population characteristics. How these trade-offs are resolved will determine which aspects of photic exposure are captured, and ongoing methodological development will continue to expand what can be practically measured in real-world settings.

## How to address field considerations with tools, assessments, and visualizations

Lighting environments are evolving into more complicated arrangements with the development of new technologies, and as our understanding of the physiological effects of light grows in parallel, being able to characterize light exposure in practice becomes increasingly important. Current recommendations are to measure and report the effective irradiance at each of the five retinal photoreceptors. This internationally balloted consensus-based method of measurement and reporting simultaneously offers both precision and flexibility^3,4^. Melanopic illuminance (standardized to equivalent daylight illuminance; melanopic EDI) weights irradiance based on the absorption spectrum of the melanopsin photopigment^4^, as the ipRGCs are primarily responsible for mediating non-visual physiological effects of light in humans^49^. Drawing from laboratory-based evidence to date, it is also recommended that, to better support human health and performance, lighting design and practice should aim for light exposures that fall within defined ranges of melanopic EDIs at particular times of day. Specifically, melanopic EDI levels for healthy young adults working standard schedules should be >250 lux during daytime hours, <10 lux in the 3 h before bed, and as dark as possible during sleep (<1 lux)^5^.

Since there is increased variability of photic exposure in field studies, a different combination of methods may be necessary to fully characterize the lighting environment as compared to lab-based research. Larger sample sizes and within-subjects study designs are more important in the field versus the lab, to account for the more varied light patterns and greater individual differences. Yet, more study participants also introduce the potential for greater variability in photic exposure. Additionally, light measurement in field settings is often more labor intensive, requiring multiple static light measurements to account for variation in light distribution across sources, space, and time. These measurements should be timed based on actual photic exposure patterns and taken from locations where individuals are most likely to be. Another consideration is the amount of time available to take light measurements, which may be quite limited in certain environments, such as hospital settings or military installations, where critical work activities must be prioritized in the space. Most research studies employing lighting interventions do not assess measures of implementation such as feasibility, practicality, or adoption of the intervention^97^. This is an unfortunate oversight, as properly conducting and reporting on an experiment may be for naught if application or adoption of the intervention proves to be impractical or impossible altogether. Therefore, basic implementation metrics should be included when conducting lighting intervention studies. With these considerations in mind, there are a variety of tools, assessments, and other methods that may be used to describe lighting environments in field settings (Table 1).

**Table 1:** Advantages and disadvantages of tools for assessing light in the field

TOOLS		PROS	CONS
**Light meters**	**General**	•Provide objective quantification of light -Some emerging technologies provide melanopic EDI as an output	•Typically more expensive than questionnaires •Staff training in use is required •Quality of instrumentation varies
	***Non-wearable light measurement devices***	•Can provide precise static photic measurements in terms of both intensity and spectral quality	•Do not account for changes in individual photic exposure patterns due to movement •Typically, do not provide continuous, high temporal resolution •Typically, should be calibrated every 1–3 years (depending on manufacturer guidelines)
	*spectroradiometer*	•High spectral resolution measures •Provides absolute measures of photic intensity and spectral power that may be used to derive alpha-opic illuminances, including melanopic EDI values	•Typically, highest in cost relative to other light meters •May require additional tools for use and/or post-processing (e.g., laptop)
	*irradiance meter*	•High quality instrumentation, typically with more precise measures of intensity relative to illuminance meters •Intensity is not weighted based on photopic (visual) system •Typically, lower in cost than spectroradiometers	•Does not provide spectral information or physiological response metrics •Typically, more expensive than illuminance meters
	*illuminance meter*	•Generally small and highly portable •Allows for quick measurements of intensity •High ease of use •Typically, lower in cost than irradiance meters and spectroradiometers •Wearable options available (see below for details)	•Intensity is weighted based on the photopic (visual) system •Does not provide spectral information and may have greater error in the short wavelength region of the spectrum
	***Wearable light devices***	•Provide continuous, high temporal resolution data •Account for changes in individual photic exposure patterns due to movement •Can be used to monitor individual photic exposure patterns in locations that are disparate, remote and/or hard to access •Each device is generally less expensive than a non-wearable meter •Some provide spectral information (e.g., RGB sensors)	•Low internal consistency between devices of the same model and insufficient calibration processes •Device options vary considerably in terms of outputs provided, making aggregation and comparison of data across different sensors difficult •Quality of data relies on compliance
**Questionnaires**	**General**	•Relatively easy to administer, process, and analyze •Lower cost than light meters •Can be scaled up relatively easily, including assessment at multiple timepoints	•Provide subjective data, which is subject to bias and may not always relate to objective measures •Do not provide continuous, high temporal resolution information in real time •Quality of data relies on visual perception, reading comprehension, and compliance
	*Harvard Light Exposure Assessment* (H-LEA)^108^	•Obtains hourly information about exposure to different light sources and thus, relatively high temporal resolution allows for tracking changes over shorter periods of time •Externally validated against objective measures	•Hourly assessment is more burdensome compared to monthly or daily assessment •Only describes source and duration, not intensity
	*Modified Consensus Sleep Diary*^107^	•Obtains daily information about perception of light surrounding sleep and thus, temporal resolution allows for tracking changes over extended periods of time •As sleep diaries or logs are already frequently used, it is easy to incorporate without significant additional participant burden	•If diaries or logs were not already being used in the study, daily assessment is more burdensome compared to monthly assessment
	*Light Exposure Behaviors Assessment (LEBA)*^109^	•Obtains prior month's information about behaviors that influence photic exposure patterns •Only known assessment (other than direct observation) that captures a comprehensive set of behaviors that influence photic exposure patterns •Single administration (or once/month) is less burdensome for participants •Reliable, internal validity	•Monthly assessment provides lower resolution information than the modified consensus sleep diary or H-LEA
**Visual Representations of a Scene**	**General**	•Helps with repeatability of future experiments •Facilitates interpretation of data, especially when there are multiple light sources and locations throughout a place of study (complicated spaces) •Allows for future integration of measurements over space and time	•Can be time consuming if drawings or models do not already exist, especially when making them accurate/to scale •Sensitive environments may not allow •They are static representations and thus, it is more difficult to reflect dynamic lighting environments
	*floor plan or reflected ceiling plan*	•Helps create measurement diagrams and plan the design of lighting interventions based on installed architectural lighting (indicated on reflected ceiling plans)	•May be difficult to obtain •Potential security concerns for certain spaces
	*diagram/schematic*	•Can help to organize and document multiple measurements at specific locations	•Time- and labor-intensive •Potential security concerns for certain spaces
	*picture*	•Real-time image/documentation of environment •Less time-consuming than other methods	•Requires a camera and ability to photograph, which may/may not be readily available •Authorization may be required •Potential security or privacy concerns for certain spaces
	*directogram*	•May be used to integrate data from multiple sources and locations, with weighting if desired	•In early stages •Correct weights are not yet known
	*software-assisted rendering*	•May be used to integrate data from multiple sources and locations, with weighting if desired	•In early stages for calculating circadian input/melanopic EDI •Best methods remain uncertain

### Light meters

Light meters quantify the physical properties of photic stimuli, including various metrics of intensity and spectral quality. In human field studies, the primary surface of interest is the eye. As irradiance at the cornea is inversely proportional to the distance squared from the light source, even minor deviations in location relative to participants’ eyes can significantly impact the accuracy of the measured dose. Thus, meter placement should approximate the position and orientation of the eye in the relevant environment.

When selecting an appropriate light measurement tool, there are often trade-offs between cost, portability, temporal resolution, spectral accuracy, and ease of use across the wide range of available light meters (see also Table 1). Light meters vary considerably in instrumentation quality and precision, and different devices provide different types of information. Measurements may be collected as single snapshots or continuously over time, with wearable light dosimeters often being used for long-term, time-resolved tracking of individual photic exposure patterns.

Devices range from benchtop instruments, which are often more stable but bulky and require external power, to portable devices that offer greater flexibility for positioning near the eye. Wearables further extend this flexibility through a range of form factors, though often with trade-offs in measurement precision.

Because nomenclature for light meters varies considerably, the CIE’s International Lighting Vocabulary (https://cie.co.at/e-ilv) is used in this manuscript for standardization purposes, though one may also consider definitions from the Illuminating Engineering Society (https://www.ies.org/standards/definitions/), the International Union of Pure and Applied Chemistry Gold Book (https://goldbook.iupac.org/), and others. Generally, the most appropriate tool will depend on the application, particularly in circadian research, where the spatial, spectral, and temporal characteristics of light exposure are critical to accurate quantification.

#### Selecting light measurement tools

No single light measurement tool can fully characterize all aspects of photic exposure that are relevant to non-visual responses in field studies. An appropriate measurement strategy will require selecting and, when necessary, combining tools based on the characteristics of the light stimuli most relevant to the study objectives. In addition, measurement limitations do not have uniform consequences; the impact of a given uncertainty depends not only on its magnitude but also on its impact within the context of the nonlinear relationship between light exposure and physiological responses.

In this section, we review characteristics of light measurement tools that should be considered when developing a measurement strategy, including temporal, intensity, spectral, and spatial measurement capabilities. We also discuss the complementary roles of light measurement devices that characterize light within specific environments and those that measure personal exposure as individuals move through different environments, here referred to as non-wearable and wearable, respectively. Finally, additional subjective assessments and complementary approaches for describing photic exposure are considered.

##### Temporal characteristics

For any given study, the required measurement frequency depends on how much light exposure is expected to vary over time and whether the timing of exposure is central to the physiological outcome. When biologically important changes in light occur between measurements, inadequate measurement frequency can misrepresent the timing, duration, and overall pattern of photic exposure, leading to inaccurate estimates of physiological effects. This is particularly important for circadian phase shifting, where light exposures of similar intensity and spectrum can produce opposite effects depending on circadian timing. For example, measuring only early-night workplace exposure while missing bright morning light during the commute home could significantly overestimate the expected phase delay. In contrast, studies conducted under stable lighting conditions or focused on acute responses during a single exposure period may require far less frequent measurement. Importantly, in populations that are heterogeneous in terms of circadian phase, such as shiftworkers, even a pulse that is delivered at a precise wall clock time can result in variable responses across individuals, as biological timing is what is critical. Wearable light dosimeters can be advantageous when the goal is to characterize personal light exposure over time, whereas non-wearable light measurement devices can provide important additional precision in information about environmental light conditions. In some field studies, combining both approaches may provide a more comprehensive characterization of photic exposure by linking individual experience with environmental conditions. Thus, measurement strategies should be designed according to the study question, along with expected temporal dynamics of both the lighting environment and the physiological outcome under study.

##### Intensity

Meter accuracy should be considered over the range of light intensities expected during the study. Equally important, however, is the biological response range. Because non-visual responses follow a sigmoidal dose-response relationship, the same magnitude of measurement error may have little effect at intensities below threshold or above saturation but significantly alter estimates of physiological response within the dynamic range of the dose-response curve. Furthermore, the biologically relevant intensity range depends on factors such as spectrum, circadian timing, and participant characteristics. For example, studies involving young children, who are extremely sensitive to light, may require accurate measurements below 5 lx^40,41^, whereas precision at these lower intensities may not be necessary for a workplace intervention study of adults. Ultimately, measurement tools should be selected based on their performance across the range of biologically relevant intensities expected for a specific study.

##### Spectrum

Spectral weighting, resolution, and calibration accuracy differ among light meters. Because non-visual responses are highly dependent on wavelength, errors in spectral characterization can significantly affect estimates of melanopic EDI and other α-opic quantities. As discussed above, action spectrum studies demonstrate that biological sensitivity varies by orders of magnitude across wavelengths, underscoring the importance of spectral characterization when estimating the biological potency of light. Although many current architectural lighting applications include polychromatic light sources with significant spectral overlap, the practical importance of spectral measurement accuracy depends on the expected spectral variability between study conditions and the precision with which biologically effective light exposure must be estimated. Spectroradiometers provide the most comprehensive characterization by measuring full SPDs, allowing for calculation of multiple α-opic metrics as well as future reanalysis as biological weighting functions evolve. Simpler devices, such as illuminance meters and colorimeters, typically provide less accurate estimates of biologically effective light exposure because they do not directly characterize the SPD.

##### Spatial configuration

Because non-visual responses depend on the angular distribution of light reaching the retina, measurement geometry should be selected to approximate that distribution. Measurement approaches that do not adequately represent the angular distribution of light reaching the eye may misestimate biologically relevant exposure and, consequently, the expected magnitude of non-visual responses. Laboratory studies suggest that retinal region of exposure can alter responses by approximately 2–3 fold, highlighting the potential consequences of measurement geometry when estimating biologically effective exposure^24–27^. Cosine-corrected irradiance measurements are commonly used to approximate light input in humans because they weight light from across a hemisphere, tapering at an angle similar to that of the human eye. In contrast, radiance (or “spot”) measurements quantify light from a specific direction or source (e.g., a display screen) and are useful when source-specific exposure is of interest but may not adequately characterize the total light incident on the eye when multiple light sources and environmental reflections contribute to the exposure. More specialized approaches, such as integrating spheres and imaging-based systems, can provide detailed characterization of spatial light distributions but are more commonly employed in controlled laboratory settings. Ultimately, cosine-corrected irradiance is generally preferred when estimating overall environmental light exposure in field settings.

##### Other practical considerations

There are a host of additional practical factors that should further guide the specific methods used to measure light for a given field study. For example, research-grade optical measurement systems generally require training, regular calibration, and careful attention to sensor positioning. Some systems, particularly those using remote sensors attached via fiber optic cables, may require additional stabilization or mounting methods to maintain a consistent angle and ensure accurate readings. Considerations such as setup time, portability, battery life, charging requirements, ease of use, and feasibility within the study setting are also important when selecting a measurement approach. The following sections describe common non-wearable and wearable light measurement devices in greater detail, including their relative advantages, limitations, and typical applications in circadian field research.

#### Non-wearable light measurement devices

##### Spectroradiometers

Spectroradiometers (sometimes referred to as spectrometers) measure the relative power at each wavelength and generate a SPD, which can then be used to calculate a wide variety of metrics (e.g., illuminance, color temperature, color rendering) (https://goldbook.iupac.org/), including alpha-opic metrics such as melanopic EDI^4^. An increasing number of spectroradiometers are starting to provide these derived outputs, and most modern devices include readings of key metrics on an LCD display, along with storage of more comprehensive data on a memory card and/or direct uploads to a compatible computer. Spectroradiometers often have special storage instructions and require calibration to a National Institute of Standards and Technology (NIST)-traceable lamp. While spectroradiometers were once quite expensive, costs have decreased considerably in recent years and are now comparable to higher end irradiance meters. Portability, spectral resolution, and intensity response need to be considered in choosing a spectroradiometer, which can all influence the cost and functionality of the device. For example, more budget-friendly options are sometimes unable to accurately measure lower light levels (e.g., <10 lux), which are still potent enough to have significant physiological effects^40,41,98^.

##### Irradiance meters

Irradiance meters measure the intensity of light in terms of radiant flux per unit area falling on a surface. In human field studies, irradiance meters should be positioned to approximate light exposure at the eye; thus, cosine-corrected optics are typically required for measuring light input. Instrumentation varies by brand and model, though most irradiance meters also require regular maintenance and calibration to a NIST-traceable lamp. If irradiance meters are the only meter type available, spectral data from a spectroradiometer is still needed to quantify biological potency. Therefore, obtaining adequate light measurements may require purchasing, renting, or borrowing a spectroradiometer, or contracting with a company to provide spectral data. The latter option may not be cost-effective in the long term but can be less expensive than purchasing a spectroradiometer for a single study.

##### Illuminance meters

Illuminance meters measure light intensity in units of photopic lux, which includes an automatic weighting based on the visual photoreceptor system. Thus, photopic illuminance (lx) does not accurately represent the biological effectiveness of light for non-visual responses. Historically, photopic illuminance has been the most reported metric, and many studies lack sufficient spectral information to derive biologically weighted measures retrospectively. Consequently, comparisons with earlier studies often continue to rely on photopic illuminance, despite its limitations. These meters are commonly used in photography and are relatively inexpensive, but do not provide the accuracy required for circadian research, nor the necessary information (spectrum, irradiance) about light; therefore, they are not a sufficient standalone meter. Most illuminance meters are not well-calibrated for measuring monochromatic or blue-enriched lights, for example. However, they may be a useful addition for spot-checking stability of a polychromatic light source or confirming dimming status, when initial illuminance measures are taken alongside those taken with more sophisticated meters.

#### Wearable light measurement devices

Wearable light measurement devices (also known as light dosimeters) are advantageous for capturing individual-level photic exposure patterns over time^99–101^. They are considerably smaller and, depending on form factor, can often be positioned in locations that better approximate the direction of gaze, such as on a lanyard, lapel, or glasses^102^. For field studies characterizing individual-level light exposure patterns, wearables are often useful and easily implementable. However, they are still most often incorporated into wrist-worn actigraphy devices, and thus if worn as intended, may not accurately reflect light reaching the eye. Further, light dosimetry requires compliance from participants in terms of consistent and continuous use, placement of the device and often, charging. Flexibility in style and placement options may increase utility across populations as well as compliance with device wear; however, lack of standardization will introduce some level of variability.

As a tradeoff for their size, portability, and cost, dosimeters do not always provide raw data, and intensity and spectral information are generally less accurate than non-wearable light meters (Table 1). Initially, light dosimeters only provided the intensity, usually in photopic lux, without spectral information. More recently, commonly used dosimeters, such as the Phillips Respironics Actiwatch Spectrum models (now discontinued but well-represented in the literature), contain three non-overlapping bands of “red”, “green”, and “blue” light (RGB) sensors. These technologies are improving rapidly, however, with multiple emerging devices that have higher-resolution spectral information from more than three channels^2,30^. While existing devices provide some spectral information, and the associated software of a limited number of companies now provide estimated melanopic EDI, most still report metrics that are weighted to reflect input to the visual rather than the circadian system. Further, dosimeters with spectral information are often more expensive than those without it; accuracy is often still poor in lower light environments (<10 photopic lux); there is low internal consistency between devices of the same model due to insufficient calibration processes; and current device options vary considerably, making aggregation and comparisons of data across different wearable light meter devices challenging^99–105^.

#### Integrating non-wearable and wearable light measurements

Together, continuous measurements from wearable devices and multiple static measurements from non-wearable light meters can provide a more complete picture of photic exposure patterns over time, space, and individuals. Wearables can be particularly helpful for assessing individual-level adherence to lighting interventions and are a useful adjunct to measurements taken with non-wearable light meters^106^; however, it has yet to be determined how to best weight and integrate those data with measures from other light measurement devices. Given the complementary strengths and weaknesses of non-wearable meters and wearable dosimeters, we recommend reporting these data types separately, while exploring ways to combine them meaningfully in post hoc analyses. Future approaches may include using static SPD measurements to calibrate wearable devices, applying contextual overlays based on room use or activity schedules, and/or incorporating both data types into predictive models rather than collapsing them into a single metric. Each approach has trade-offs and feasibility considerations that depend on the setting, available data, and research question.

### Subjective assessments

Surveys, diaries, and questionnaires generally require less cost and expertise to utilize as compared to light meters, and they also have the advantage of being relatively easy to administer across larger samples of participants in geographically diverse locations. Information about visible light in an environment over time, sleep patterns, and other behaviors that influence light exposure patterns may be assessed via validated questionnaires, such as the modified Consensus Sleep Diary^107^, Harvard Light Exposure Assessment (H-LEA)^108^, and the Light Exposure Behaviors Assessment (LEBA)^109^. For example, adding a few questions about light exposure in the sleeping environment to a daily diary is a low-burden way to collect information about light at the time(s) it is likely to have the largest impact on sleep and circadian health^107^. Similarly, validated questionnaires about the types of light sources experienced throughout the day^108^ or light exposure behaviors across the month^109^ provide more in-depth data across time. Additionally, surveys can be used to obtain relevant information about sample characteristics that can influence light exposure, including age, chronotype, eye health, color vision status, photosensitivity, vision impairment, and use of medications. Feelings and symptoms attributed to lighting (e.g., headache, glare, mood changes), as well as how lighting is experienced (e.g., perceived brightness, comfort, satisfaction), the ease and likelihood of future use, and any barriers to implementation and intervention adherence are also helpful pieces of information to obtain since they may influence light exposure patterns and can be used to facilitate behavior modification and adoption of novel lighting technologies^90,92,97^. Opportunities for more open-ended feedback, such as via post-intervention interviews (informal or structured) with management and participants may also help identify barriers to implementation^97^.

While a significant amount of data can be obtained via questionnaires, subjective assessments introduce several limitations. They increase the potential for bias, depend heavily on participant compliance, can lead to missing data, and place a higher burden on participants and staff relative to passive measures like wearables. Additional measures and further validation of recently developed survey tools are also needed. Nonetheless, these existing tools remain highly useful for providing preliminary information or augmenting objective light data.

### Visual representations

Because spatial arrangement of photic stimuli is much harder to measure and control for in field studies than lab studies, visual representations such as diagrams, pictures, schematics, and renderings of lighting environments may be key for interpretation of findings and replicability^30^. These tools can be created via a variety of mediums, including photographs, illustrated schematic representations, and/or software-assisted renderings. For example, a diagram or reflected ceiling plan may be quite useful for recording multiple measurements or for noting areas of high traffic/use, whereas a schematic, directogram, or other visual representation, perhaps with the aid of software, are helpful for integrating and/or conveying that information in a meaningful way. Models and software are likely to continue being developed, tested, and refined, which will aid in the integration of multiple measurements across dynamic lighting environments.

Table 2 provides a more comprehensive understanding of how to apply the tools enumerated in Table 1 (i.e., light meters, subjective assessments, visual representations) in the context of the previously described pre-ocular, ocular, and post-ocular factors that influence light exposure in the field, along with additional methods for addressing those characteristics.

**Table 2:** Recommended methods for assessing and addressing the various characteristics that influence photic exposure and the physiological effects of light

## Concluding remarks

As our understanding of the physiological effects of light has grown, so have available lighting technologies. Because of both, the variability of the photic environment has increased and will likely only continue to do so. With innovations in spectral engineering, LEDs can now be tuned to optimize visual functions, such as color rendering, as well as other non-visual physiological effects, such as circadian responses^110^. Because LEDs can be manipulated to suit various environments with unique considerations, they will be particularly helpful in providing novel integrated lighting solutions that better support human health and well being^1^. However, there remain many research questions and gaps in understanding how the physiological effects of light are influenced at the pre-ocular, ocular, and post-ocular level in real world settings, and it is difficult to prioritize certain factors over others without existing evidence to guide us. Recently published recommendations for the laboratory are a good place to start, with additional emphasis on describing the population and environment(s) to the greatest extent possible, since these study elements often cannot be controlled in the field in the same way they typically are in the lab. Implementation measures provide insight into utility and likely uptake of specific lighting solutions, and they are useful and easy to integrate into intervention studies moving forward^97^.

**Fig. 3:**
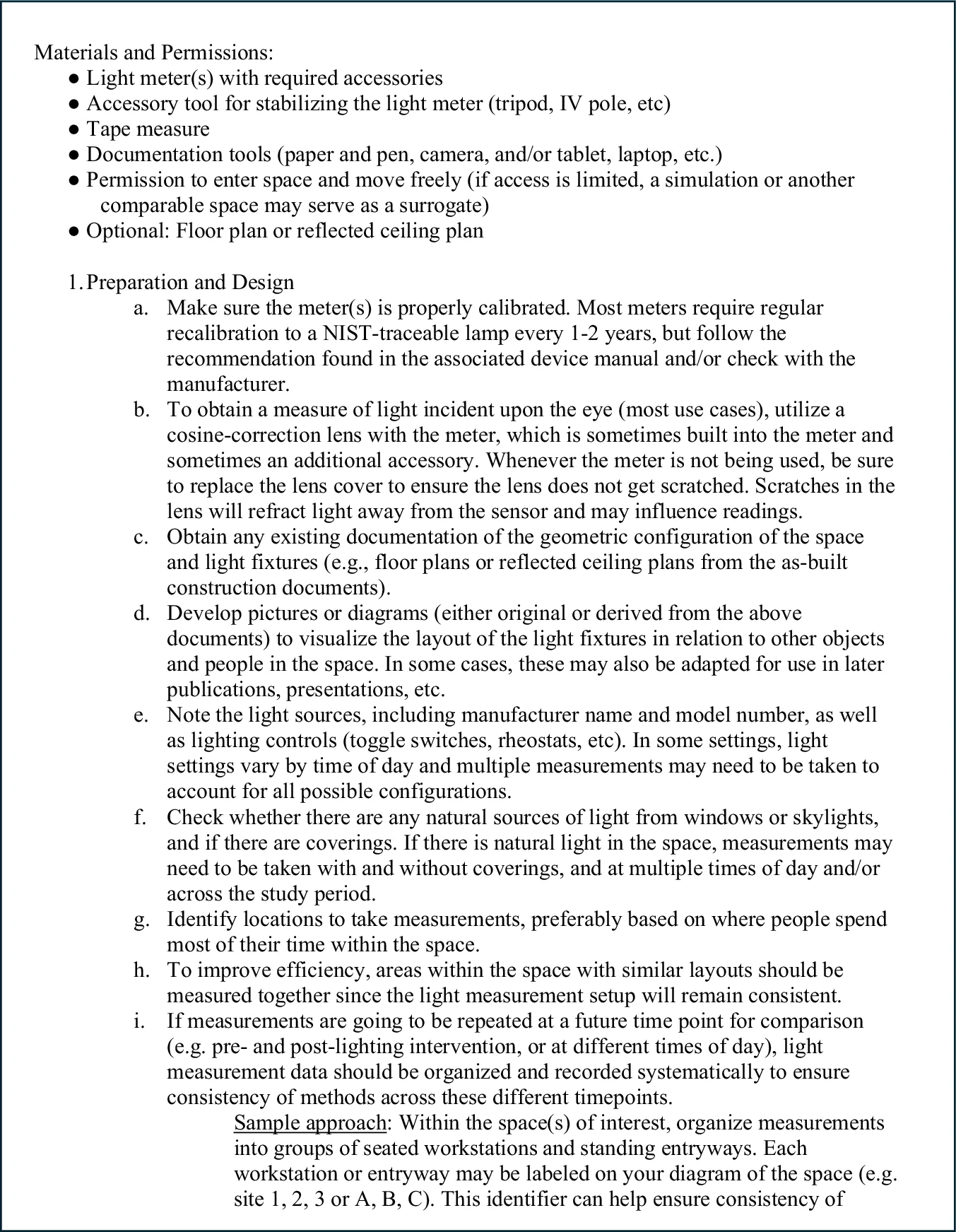

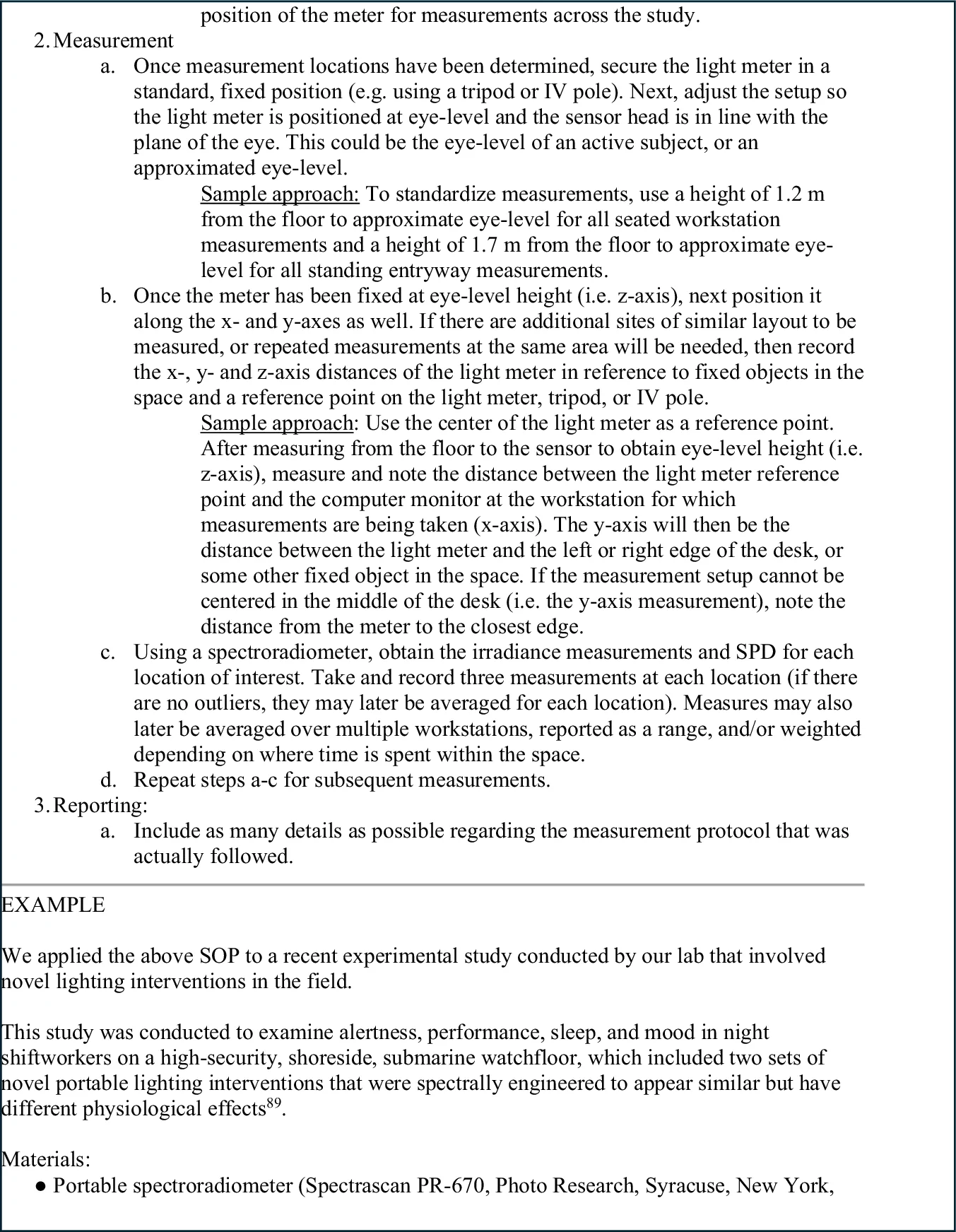

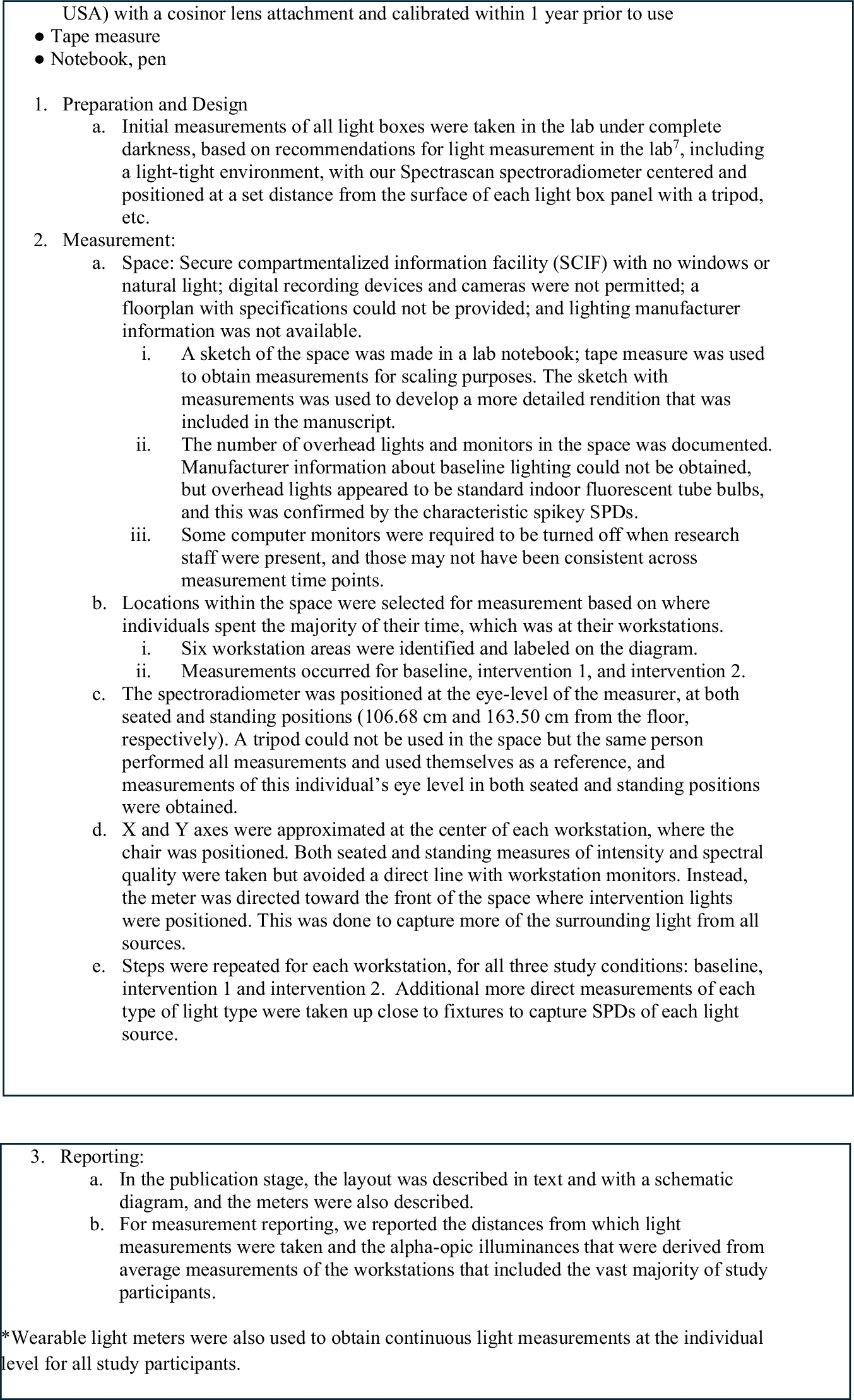
Standard operating procedure (SOP) for light measurement in the field. Here we provide an SOP for light measurement in field studies, along with an example for how the SOP has been applied in a recent lighting intervention study on a high-security watch floor conducted by our lab^92^.

Advances will continue to develop with not only lighting technologies, but also with meters, sensors, and software, which will aid in measuring, mapping, integrating, and reporting light throughout a space. In the meantime, we have provided a standard operating procedure (SOP; Fig. 3) that can be used to guide the process of light measurement in field studies, along with an example to demonstrate how the SOP has been applied in our own research, with the caveat that it is still a work in progress. The primary principle is to measure as many relevant factors as possible, as well as possible. If resources are limited, less preferred but practical options include borrowing, sharing, or renting more expensive equipment that is not needed regularly (e.g., spectroradiometers for obtaining SPDs of all light sources in a space). Wearables are useful for providing near-continuous measurements, but when possible, should be used in conjunction with multiple static measurements and reported separately. If both irradiance and SPDs can be obtained- either measured in-house (preferably) or obtained from the manufacturer (less ideal)- then alpha-opic illuminances may be estimated using available toolkits^4^. Lastly, reasons for why deviations from ideal practices were made should be reported (e.g., inability to obtain comprehensive measurements in the actual environment because it would be too disruptive to operations, and therefore prioritizing spectral resolution and irradiance measures in the highest traffic areas and supplementing them with measurements estimated in a simulation of the space). Ultimately, optimizing measurement and reporting of light in field studies will improve interpretation of findings, facilitate replication and comparability across future studies, and accelerate the development of effective, integrated lighting solutions that support human health and performance.

## Data Availability

No datasets were generated or analyzed during the current study.
